# Highly sensitive optical ion sensor with ionic liquid-based colorimetric membrane/photonic crystal hybrid structure

**DOI:** 10.1038/s41598-020-73858-8

**Published:** 2020-10-07

**Authors:** Daiki Kawasaki, Ryoutarou Oishi, Nao Kobayashi, Tatsumi Mizuta, Kenji Sueyoshi, Hideaki Hisamoto, Tatsuro Endo

**Affiliations:** 1 Osaka Prefecture University, Sakai, Japan; 2 JST PRESTO, Saitama, Japan

**Keywords:** Sensors, Lab-on-a-chip

## Abstract

An ionic liquid-based thin (~ 1 µm) colorimetric membrane (CM) is a key nano-tool for optical ion sensing, and a two-dimensional photonic crystal slab (PCS) is an important nano-platform for ultimate light control. For highly sensitive optical ion sensing, this report proposes a hybrid of these two optical nano-elements, namely, a CM/PCS hybrid. This structure was successfully fabricated by a simple and rapid process using nanoimprinting and spin-coating, which enabled control of the CM thickness. Optical characterization of the hybrid structure was conducted by optical measurement and simulation of the reflection spectrum, indicating that the light confined in the holes of the PCS was drastically absorbed by the CM when the spectrum overlapped with the absorption spectrum of the CM. This optical property obtained by the hybridization of CM and PCS enabled drastic improvement in the absorption sensitivity in Ca ion sensing, by ca. 78 times compared to that without PCS. Experimental and simulated investigation of the relation between the CM thickness and absorption sensitivity enhancement suggested that the controlled light in the PCS enhanced the absorption cross-section of the dye molecules within the CM based on the enhanced local density of states. This highly sensitive optical ion sensor is expected to be applied for micro-scale bio-analysis like cell-dynamics based on reflectometric Ca ion detection.

## Introduction

Ionic liquid (IL) is a unique molten salt, which possesses a low melting point below 100°C^1,2^. IL has various unique properties, such as high thermal stability, high chemical stability, non-volatility, and high conductivity^3,4^. Recently, IL has been applied to liquid materials for analytical chemistry due to their unique and tunable properties^4–9^.

Plasticized poly(vinyl chloride) (PVC) membranes have been used for optical chemical sensors (optodes). An optode spectrally and visually shows a color change due to the structural change of an indicator dye in a membrane, which is caused by the extraction equilibria of ion species between the membrane and sample solutions^10–14^. Thus, the sensitivity is generally determined by the dye concentration and membrane thickness according to the Lambert–Beer law. On the other hand, we recently developed an IL-based colorimetric membrane (IL-CM), which is composed of a lipophilic IL-based dye acting as a colorimetric plasticizer^8,15–19^. The IL-CM contains an extremely high concentration of dye in a thinner membrane (< 1 μm), realizing extreme sensitivity and response speed. However, the sensitivity is still too low to analyze or detect ions in a micro-scale area by microspectroscopy, for example, in vitro optically real-time observation of cell dynamics based on Ca ion flux detection^20–23^. Thus, considerable sensitivity improvement is necessary to advance the application of IL-CMs, such as in chemical bio-imaging tools, which demand high signal-to-noise ratios and spatial resolutions. Consequently, a novel optical system that can break the sensitivity limit attributed to the Lambert–Beer law must be developed.

Photonic crystals (PhCs) enable the spatial and temporal control of light as photonic band-based optical cavities^24–26^. The highly localized light with a strongly enhanced electric field generated by a PhC considerably enhances light–matter interactions; hence, PhCs have been applied to enhance spontaneous emission, such as in fluorescence enhancement and lasing^27–30^. A two-dimensional photonic crystal slab (PCS) confines light, re-emitting it into free space or enhancing the light–matter interaction. Due to their optical properties, PCSs have been theoretically and practically studied in various research fields, such as surface-emitting lasers, energy conversion, and optical sensors^31–37^. In optical ion sensor application, photonic crystal based on hydrogel or cholesteric liquid crystal (CLC), of which mechanism to detect ions was structural change with ion extraction into those materials, have been investigated. These approaches enabled high-sensitivity but was not suitable for micro-scale measurement and observation^38,39^. On the other hand, PhC-microcavity laser with ion-responsive polymer layer successfully achieved highly-sensitive micro-scale Ca ion detection by wavelength shift as signal change. however, using infrared light, it could not be applied into microscopic observation with visible light^40^. Photonic crystal fiber-based sensors have been well investigated and applied in bio-sensing, however, they would be also hard to be incorporated into observation system^41–44^. We have also applied PCSs to various optical sensors, such as label-free immunosensors and absorptiometric and fluorometric ion sensors^45–50^.

PhCs considerably enhance the absorption or emission efficiencies of excitonic matter. The former has often been studied for absorption saturation, energy conversion efficiency enhancement, and up-conversion luminescence^51–57^, while the latter has been investigated for spontaneous emission enhancement^27–30^. Focusing on the former, it is expected that the absorption efficiencies of dye molecules can be drastically improved due to the PCS-enhanced local density of states (LDOS). Thus, PCSs are expected to improve the absorption sensitivity of IL-CMs substantially when the absorption spectrum of the dye molecules and reflection spectrum of the PCS overlap, and spectral tuning is easily achievable according to the design of the PCS structure. Therefore, this approach does not require any design of the IL-CM of the constituent dye molecules.

**Figure 1:**
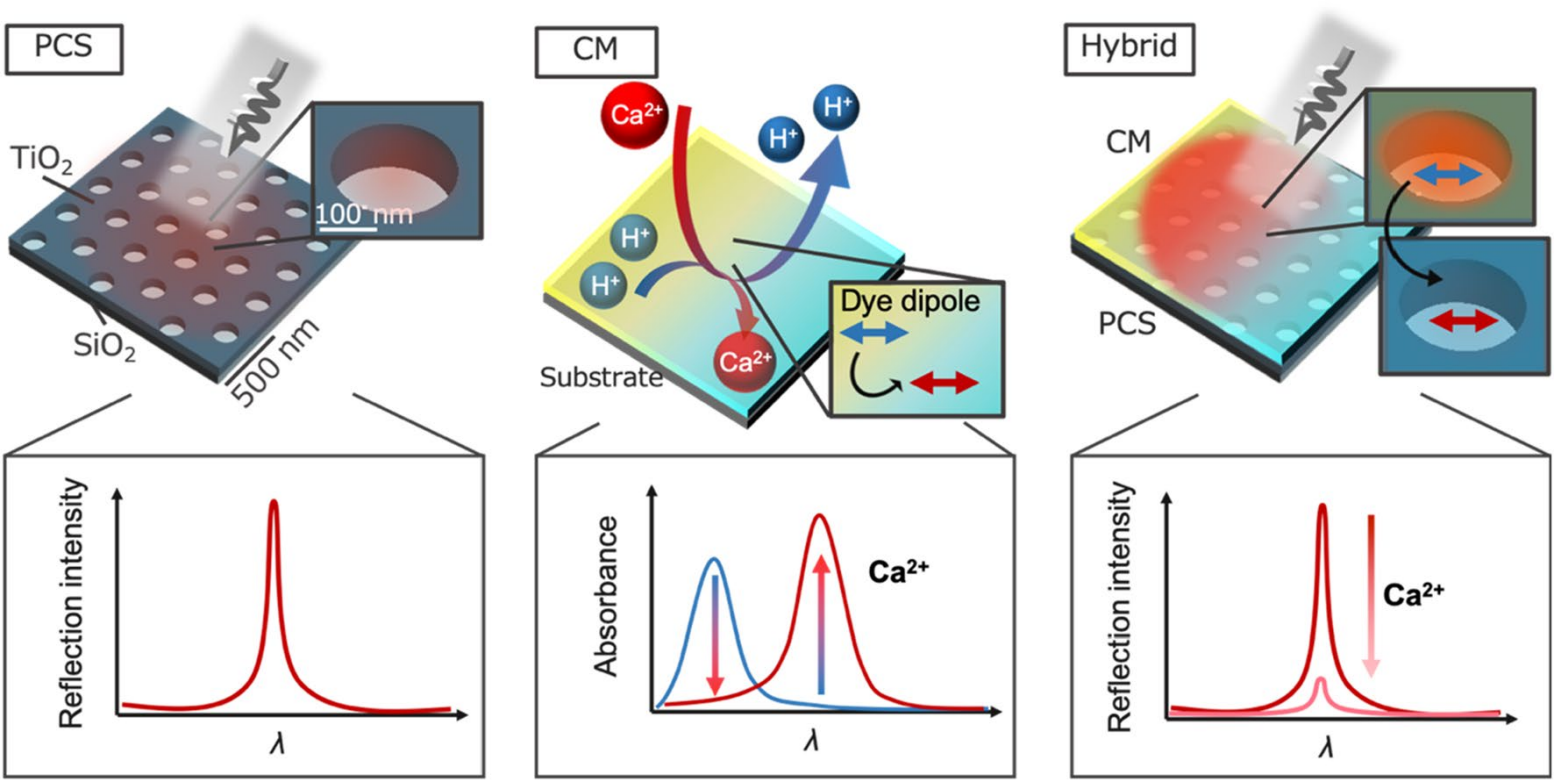
Schematic illustrations of the PCS, CM, and CM/PCS hybrid, as well as their optical characteristics. The PCS emission wavelength overlaps with the CM absorption wavelength when the CM extracts Ca^2^^+^ and releases protons. Thus, the emission intensity is decreased substantially by extracting Ca^2^^+^ using the CM because the dye molecules absorb the light localized in the PCS.

In this study, we designed a hybrid nanostructure composed of an IL-based Ca ion-responsive CM and a titanium dioxide (TiO_2_)-based PCS (CM/PCS hybrid). Figure 1 provides schematic illustrations of the PCS, CM, and hybrid structure, as well as their optical properties. In this work, hybrids with various thicknesses of CM were successfully fabricated, and their optical properties were characterized. The correlation between the absorption sensitivity and thickness of the CM of the hybrid structure was evaluated by optical measurement of the Ca ions. The experimental results and electromagnetic calculations based on the finite-difference time-domain (FDTD) method demonstrated that the absorption sensitivity of the CM with PCS was drastically improved based on the absorption cross-section enhancement due to the high LDOS, and the absorption was enhanced by up to ca. 78 times compared to that of the original CM without a PCS.

## Results

### Fabrication of the CM/PCS hybrid

**Figure 2:**
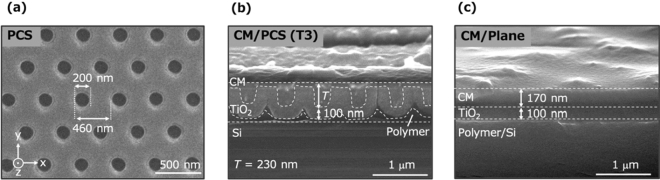
SEM images of (**a**) PCS surface, (**b**) CM/PCS hybrid (T_3_) cross-section, and (**c**) CM/Plane cross-section. In (**a**), the lattice constant of the PCS is 460 nm, the hole diameter is 200 nm, and the SEM image shows that the PCS is crack-free. The scale bar represents 500 nm. In (**b**), the thickness of the TiO_2_ bulk of the PCS is 100 nm, the thickness of the CM (T_3_) is 230 nm, and the polymer layer is used to attach the TiO_2_ layer to the substrate. In (**c**), the thickness of the CM is 170 nm. The scale bars in (**b**) and (**c**) represent 1 μm.

CM/PCS hybrid structure is composed of an IL-CM and a TiO_2_-based PCS, as shown in Fig. 1. Figure 2a presents a scanning electron microscopy (SEM) image of the TiO_2_-based PCS. The lattice constant is 460 nm, the hole diameter and depth are 200 nm, and the TiO_2_ bulk thickness is 100 nm (Fig. 2a,b). By spin-coating the solution including the CM components and solvents onto the PCS, CM/PCS hybrids with four CM thicknesses, 50 (T_1_), 100 (T_2_), 230 (T_3_), and 300 (T_4_) nm, were fabricated. A hybrid structure composed of the CM (170 nm thickness) and TiO_2_-based plane slab (CM/Plane hybrid) was also fabricated for comparison. The thickness of the CM on the PCS was defined as the distance from the bottom of the PCS hole to the surface of the CM. The CM thickness was successfully controlled by changing the dilution rate of the CM components in the solvents. Figure 2b,c shows cross-sectional scanning electron microscopy (SEM) images of the CM/PCS hybrid structure with a 230-nm-thick CM and the CM/Plane hybrid, respectively. Figure S1 provides SEM images of the other hybrid structures. To observe the structures by SEM, a Si substrate was used instead of the SiO_2_ substrate that is commonly used in experiments. The fabrication process is described in the methods section and schematically shown in Fig. S2.

### Optical characterization of the PCS and CM/PCS hybrid

**Figure 3:**
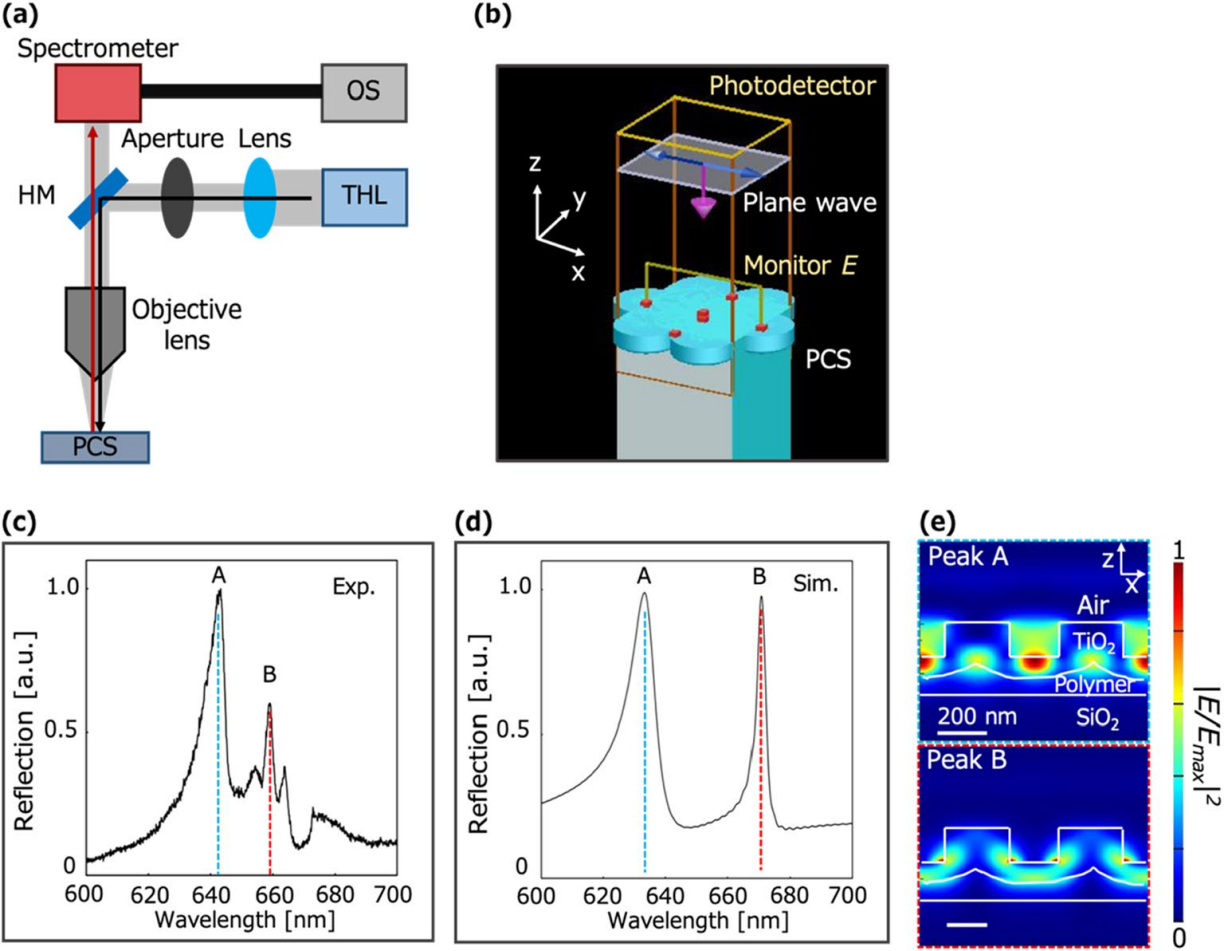
Optical characterization of the PCS. (**a**) Schematic of the optical setup used to measure the reflection spectra of the PCS. THL: Tungsten-halogen lamp, HM: Half-mirror, OS: operating software. A long-working-distance objective lens (×10) was used. The spot diameter was 250 μm. (**b**) Simulation model for optical characterization of the PCS. The plane wave was cross-polarized. A photodetector measured the reflection spectra, and Monitor *E* calculated the electric field distribution. (**c**) Experimental reflection spectrum of PCS in air. The peak wavelengths are 641 nm (A, blue dashed line) and 660 nm (B, red dashed line). (**d**) Calculated reflection spectrum of PCS in air. The peak wavelengths are 637 nm (A) and 668 nm (B). (**e**) Calculated electric field distributions of PCS at the peak wavelengths of A and B, respectively. At the peak A wavelength, the light is localized in the holes of the PCS (mode A), while at the peak B wavelength, it is localized in the TiO_2_ bulk. *E*_*max*_ is the maximum electric field intensity (mode B). The scale bars represent 200 nm.

The optical properties of the PCS were characterized by measuring the reflection spectrum under an air atmosphere using a home-made optical setup (Fig. 3a) and an electromagnetic simulation of the PCS structural model based on FDTD calculations (Fig. 3b). Figure 3c, d presents the experimental and simulated reflection spectra, respectively, confirming the existence of two PCS modes (modes A and B). The experimentally obtained peak wavelengths of modes A and B were 641 nm and 660 nm, respectively, while their simulated wavelengths were 637 nm and 669 nm, respectively. The quality factors *Q* of modes A and B were 163 and 412, respectively, in the experimental results and 141 and 409, respectively, in the simulation results (*Q* = *λ*_peak._/FWHM, *λ*_peak_: peak wavelength, FWHM: full width at half-maximum). The incident light was not polarized in the experiment, while it was polarized in the simulation. It was confirmed that the polarization of the incident light had little effect on the reflection spectra of the PCS. The slight differences of the peak wavelengths between the experiment and simulation were attributed to the structural differences caused by fabrication errors and structural heterogeneity. The mode distributions generated by the PCS at the peak wavelengths were calculated. In mode A, the light was localized in the holes of the PCS, while in mode B, it was localized in the TiO_2_ slab (Fig. 3e). These resonance modes generated enhanced electric fields, which were expected to increase the LDOS. In this work, we focused on mode A because the absorption efficiency of the dye molecules in the holes of the PCS was expected to be enhanced.

**Figure 4:**
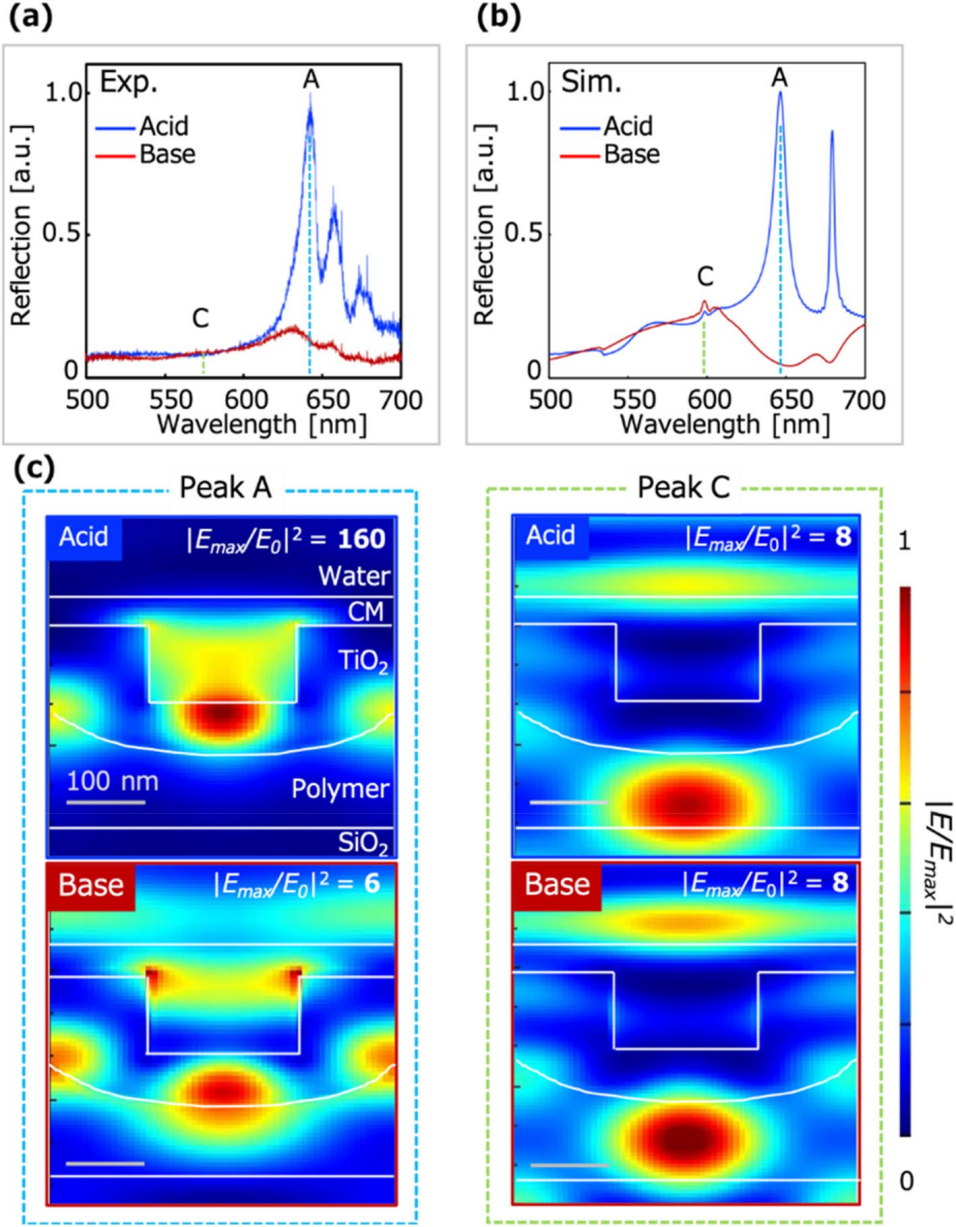
Optical characterization of the CM/PCS hybrid (T_3_). (**a**, **b**) Reflection spectra of the CM/PCS hybrid immersed in acidic (blue) and basic (red) solutions observed (**a**) experimentally and (**b**) in the simulation. The wavelengths of peaks A (blue dashed line) and C (green dashed line) are 642 nm and 573 nm, respectively, in the experimental results and 647 nm and 582 nm, respectively, in the simulation results. The reflection intensity of peak A is drastically decreased while that of peak C is slightly increased due to immersion of the CM/PCS hybrid in the basic solution. (**c**) Calculated electric field distributions of the CM/PCS hybrid at the peak wavelengths of modes A and C. The electric field enhancement factor *|E*_*max*_/*E*_0_|^2^ is stated in each figure, and the normalized electric field enhancement factor |*E*/*E*_*max*_|^2^ is shown by the color distribution. It is decreased by 96% in mode A but slightly increased in mode C. In mode A, the localized light decays due to the extinction coefficient of the CM, while in mode C, it is only slightly affected by the CM. The scale bars indicate 100 nm.

The optical properties of the CM/PCS hybrid (T_3_) were characterized, as described in this section. The optical responses of the hybrid to acidic (0.1 M phosphoric acid (H_3_PO_4_)) and basic (0.1 M sodium hydroxide (NaOH)) solutions, which corresponded to complete protonation and deprotonation of the dye in the CM, were analyzed by experimental measurement of the reflection spectra and electromagnetic simulation. The simulation model was constructed based on the fabricated hybrid structure. Figure 4a, b presents the experimental and calculated reflection spectra, respectively, of the CM/PCS hybrid immersed in the acidic and basic solutions. In the case of the Ca ion response shown in Fig. S3, the extinction coefficient *κ* in the wavelength range of 500–800 nm increases as the dye molecules in CM are deprotonated. *κ* was calculated based on the experimental results in our previous work^18^. Then, the reflection intensity decreases due to the absorption of localized light in the PCS by the CM. The reflection intensity of peak A decreases, while that of peak C slightly increases with immersion of the CM/PCS hybrid in the basic solution. According to the calculated electric field distributions in mode A, the localized light in the hole drastically decayed, which is expected to be due to the increase in the extinction coefficient of the CM. The maximum electric field enhancement factor *|E*_*max*_/*E*_0_|^2^ is decreased by 96%, where *E*_0_ is the incident light intensity. On the other hand, the electric field distribution at peak C (mode C) shows that the light is localized in the CM when the CM/PCS hybrid is immersed in acidic solution, and its intensity is slightly increased in basic solution, resulting in the slight increase in the reflection (Fig. 4c). These results suggest that mode A enhances the absorption cross-section of the dye molecules in the CM. The average peak wavelength of the CM/PCS hybrids (T_1_–T_4_) is 641.8 nm, and the peak wavelengths do not depend on the CM thickness. The peak intensities also are not thickness-dependent (Fig. S4). Thus, when the hybrid is immersed in acidic solution, the effect of the CM thickness on the PCS optical properties need not be considered.

### Response of the CM/PCS hybrid to Ca ions

**Figure 5:**
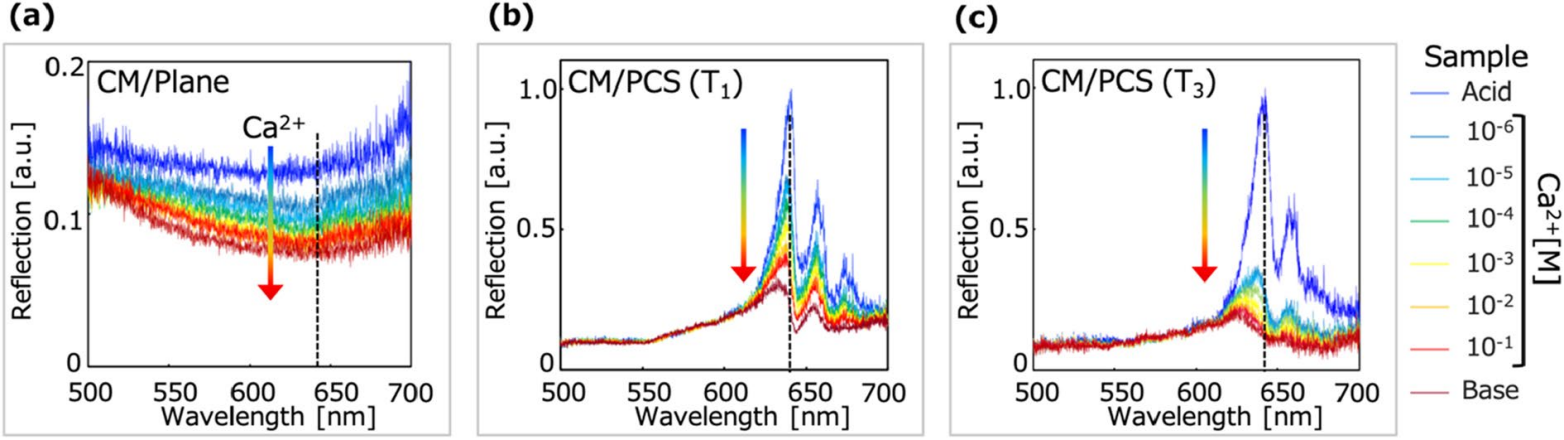
Reflection spectrum changes of the CM/PCS hybrid due to the response to calcium ions. (**a**) Reflection spectrum change of the CM/Plane hybrid. The reflection intensity at a wavelength of 640 nm (black dashed line) was analyzed. The rainbow-colored arrow indicates the increment of Ca ions. As Ca ions concentration was increased and extracted into the CM, absorption intensity was increased and then, reflection intensity was decreased (blue to red plots). (**b**, **c**) Reflection spectra of the CM/PCS hybrid (T_1_ and T_3_). The reflection intensity at the wavelength of the peak intensity for the acidic solution was analyzed. The reflection intensity was also decreased with the increment of Ca ions. The reflection intensity change was different from each thickness of the CM.

**Figure 6:**
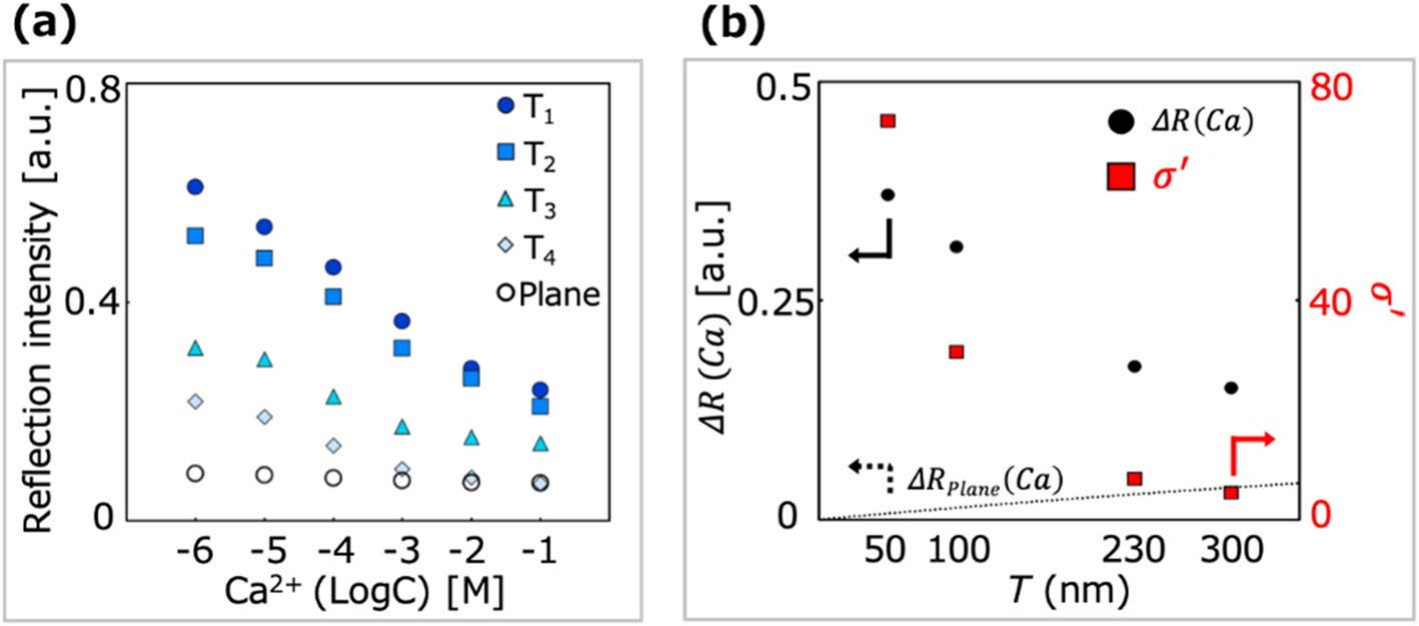
Response of the CM/PCS hybrid to calcium ions. (**a**) Reflection intensities of the hybrid structures for different calcium ion concentrations. As shown in Fig. 5, the increment of Ca ions concentration caused the decrement of reflection intensity of the CM/PCS (T_1_–T_4_) and the CM/Plane structures. The plotted values are the average values of triplicate experimental results (N = 3). (**b**) Reflection intensity change as the responsivity of the CM/PCS hybrid structure to Ca ions in the range of 10^−6^–10^−1^ M, which was described as the reflection intensity change from the reflection intensity of Ca ions concentration of 10^−6^ M to 10^−1^ M, $\Delta R\left( {Ca} \right)$ (black circles). Reflection intensity change of the CM/Plane hybrid structure $\Delta R_{Plane} \left( {Ca} \right)$, which was estimated from experimentally obtained results when CM thickness was 170 nm (see Text S1), (dashed line); and absorption enhancement factor, $\sigma ^{\prime} = \sigma_{PCS} /\sigma_{Plane}$, where $\sigma_{PCS}$ and $\sigma_{Plane}$ describes the absorption intensity per unit of the CM thickness, $\sigma = \Delta R\left( {Ca} \right)/T$, obtained by the CM/PCS and the CM/ Plane, respectively (red squares).

This section describes the responses of the CM/PCS hybrids and CM/Plane hybrid to Ca ions (10^−6^–10^−1^ M in Tris–HCl buffer, pH 7.0), which were investigated to evaluate the enhancement of the absorption sensitivity by comparing the CMs with and without the PCS. Figure 5 depicts the reflection spectrum changes of the CM/Plane and CM/PCS (T_1_ and T_3_) hybrid structures due to the response to Ca ions. The reflection intensity decreases as the Ca ion concentration increases due to the increment of the light absorption by dye molecules in the CM. Figure 6a shows a plot of the reflection intensities for each sample solution. These results indicate that the CM/PCS hybrid responds to Ca ions, and that the responsiveness depends on the thickness of the CM. The reflection intensity for the concentration of Ca ions C [M] was defined as $R\left( {LogC} \right)$, and the reflection intensity change as the responsivity to Ca ions, ${\Delta }R\left( {Ca} \right)$ was defined as

1 $$ \begin{array}{*{20}c} {\Delta R\left( {Ca} \right) = R\left( { - 6} \right) - R\left( { - 1} \right)} \\ \end{array} $$

The absorption intensity per unit thickness *σ* [nm^−1^] and absorption enhancement factor *σ*′ were defined as

2 $$ \begin{array}{*{20}c} {\sigma = \Delta R\left( {Ca} \right)/T} \\ \end{array} $$

3 $$ \begin{array}{*{20}c} {\sigma ^{\prime} = \sigma_{PCS} /\sigma_{Plane} } \\ \end{array} $$

where *T* [nm] is the CM thickness, $\sigma_{PCS}$ describes $\sigma$ of the CM/PCS hybrid, and ${\upsigma }_{Plane}$ describes $\sigma$ of the CM/Plane hybrid. Figure 6b presents the experimentally obtained ${\Delta }R\left( {Ca} \right)$ and $\sigma ^{\prime}$ for each CM thickness. Against our expectation that the CM/PCS hybrids with thicker CMs would show greater reflection intensity changes as the absorption intensity increases with the quantity of dye molecules, ${\Delta }R\left( {Ca} \right)$ decreases with increasing *T*. $\Delta R_{Plane} \left( {Ca} \right)$ was estimated based on the experimental results obtained using the CM/Plane hybrid structure according to the Eq. 1. The CM/Plane hybrid shows a reflection intensity change $\Delta R_{Plane} \left( {Ca} \right)$ of 0.017 when the CM is 170 nm thick. Then, the reflection intensity change was estimated according to the Lambert–Beer law (see Text S1, eq. S9). Meanwhile, as expected, $\sigma ^{\prime}$ drastically increases with decreasing *T*, resulting in a maximum value of ca. 78 for 50 nm thickness (T_1_). These results suggest that the absorption cross-section of dye molecules depends on the LDOS-enhancement due to the enhanced electric field generated by the PCS (Fig. 3e, peak A). The absorption cross-sections of the dye molecules were expected to decrease with increasing distance from the bottom of the PCS holes because the enhanced electric field decayed exponentially from the bottom of the holes to the free space above the PCS surface. To confirm the contribution of LDOS enhancement to the increase in the absorption sensitivity of the CM, the theoretical responsivity ${\Delta }R_{model} \left( {Ca} \right)$ and absorption enhancement factor $\sigma ^{\prime}_{model}$ were calculated based on the PCS-enhanced LDOS obtained by the FDTD calculation, according to the Eqs. (1) and (3), respectively. These results agreed well with the experimental results and theoretical calculations. These findings suggest that the theoretical thickness of the CM that produces the maximum responsivity to Ca ions is 70 nm (Text S1, Fig. S5) and show that the PCS-enhanced LDOS is attributable to the enhanced absorption cross-section of the dye molecules, which leads to the drastic improvement of the responsivity to Ca ions. The theoretical calculations suggest that the optimal CM thickness is given by the relation between the extinction of incidental light and absorption enhancement. The extinction of incidental light causes the PCS mode excitation intensity to decrease, which decreases the responsivity, and the extinction of incidental light increases with increasing thickness. Meanwhile, the absorption enhancement increases with decreasing thickness, which enhances the responsivity. The optimized CM thickness improves the responsivity the most. In addition, the responsiveness of the CM /PCS hybrid (T_3_) to other cations, Mg^2+^, Na^+^, and K^+^, was investigated (Fig. S6). It was observed that the CM/PCS hybrid selectively responds to Ca ions. Moreover, the difference between Ca and Mg ions in the low concentration range of 10^−6^–10^−4^ M was found to be much more remarkable than that shown in the previous work^18^. This finding confirms that the sensitivity to Ca ions can be drastically improved by hybridizing the CM with the PCS, resulting in apparent improvement of the selectivity for Ca ions.

## Discussion

In this study, we demonstrated drastic improvement of the absorption sensitivity of a CM/PCS hybrid system, which enhanced the absorption cross-section of the dye molecules based on the LDOS enhancement by the PCS. The CM/PCS hybrid was fabricated by spin-coating the CM onto the PCS, and the thickness of the CM on the PCS was controllable by changing the dilution rate of the CM components in the solvents. The PCS and CM/PCS hybrid were optically characterized by the experimental results and FDTD-based simulation. When the PCS was hybridized with the CM, the light confined in the holes was absorbed by the dye molecules in the CM due to the spectral overlap between the PCS reflection and dye molecule absorption. The responsivity of the CM to Ca ions was drastically improved by the hybrid system, and the absorption cross-section of the dye molecules was considerably enhanced with increasing LDOS in the PCS. The maximum enhancement factor was 78.0 when the CM thickness was 50 nm, which was the smallest thickness in the experimental demonstration. The theoretical calculations suggested that the absorption enhancement would exponentially increase with decreasing CM thickness, while the optimal CM thickness (70 nm) would maximize the responsivity. This approach enables CM absorption sensitivity improvement without any design of the molecules or a membrane to enhance its absorption efficiency, but rather only by designing the photonic crystal structure so that the spectra overlap. Moreover, we achieved two important aspects for micro-scale bio-imaging. One aspect is a reduced CM thickness by one-fourth compared to that of the original CM in our previous work, which leads to much shorter response time to cations than that of pristine CM without degrading the responsivity and sensitivity. The other aspect is improve the sensitivity with micro-spatial resolution (spot diameter: 250 μm), which is much smaller than in conventional absorptiometry^18^. As the response time and spatial resolution were not investigated in detail in this study, we should evaluate these aspects in future research to enable application of the hybrid structure to chemical bio-imaging, such as that used for cell analysis. It should also be mentioned that the proposed mechanism, which enhances the absorption efficiency of the molecules or membrane, is expected to be applied to enhance molecular absorption in various fields, such as catalysis, light harvesting, and photosynthesis, as well as absorptiometry.

## Method

### PCS fabrication

The TiO_2_-based PCS was fabricated by liquid phase deposition (LPD) and nanoimprint lithography^46^. Cycloolefin polymer (COP)-based nano-pillar array structure film, which was used as a mold, was washed with ethanol and ultra-pure water. The surface was then modified by atmospheric plasma treatment for 5 min. The COP mold was filled with LPD solution, which was a mixture solution of diammonium hexafluorotitanate [0.15 M, (NH_4_)_2_TiF_6_], boric acid (0.45 M, H_3_BO_3_), and hydrochloric acid to adjust the pH to approximately 3.0, at 40 °C for 90 min. Then, 100 nm-thick TiO_2_-layer on the COP mold was obtained, and it was washed with water. After TiO_2_ deposition, the TiO_2_ layer was attached to the glass substrate using photo-curable resin, followed by dissolution of the COP mold with D-limonene. Then, the PCS was obtained (Fig. S1). To tune the optical properties of the PCS so that the reflection peak wavelength matched to the absorption of the CM, the TiO_2_-thickness was tuned to 100 nm by controlling LPD time. The correlation between the TiO_2_-thickness and LPD time have been investigated, and then LPD time was determined as 90 min. The COP mold (FLP230/200-120, pillar diameter and distance: 230 nm, height: 200 nm) was purchased from Scivax Co. Ltd. (Kanagawa, Japan). Diammonium hexafluorotitanate, ethanol, boric acid, and D-limonene were purchased from Fujifilm Wako Pure Chemical Corporation (Osaka, Japan). The photo-curable polymer (NOA81) was purchased from Norland Products Inc. (Cranbury, USA).

### CM/PCS hybrid structure fabrication

First, colorimetric Ca-responsive IL-based dye ([KD-M13][OP_2_P]) was prepared according to the method reported by our group previously^18^. Poly(vinyl chloride) (PVC) (10 mg) and [KD-M13][OP_2_P] (90 mg) were dissolved in a 5 mL mixture of ethanol/tetrahydrofuran (THF, 1:4 (v:v)). In addition, to control the thickness of the CM based on the different volatility of each mixture with different THF concentrations, the cocktail was diluted 2, 4, and 8 times by the same mixture of ethanol/THF. Then, the four kinds of cocktails prepared were spin-coated at 2000 rpm for 30 s onto the surface of the separate PCSs, and the hybrid structures with different thicknesses *T* of the IL-based CM (*T* = 50, 100, 230, and 300 nm; Fig. S2) were obtained. In the same way, the CM/Plane hybrid structure (*T* = 170 nm) was fabricated as the control. Bis(4-*n*-octylphenyl)phosphate Ca salt was purchased from Dojindo Laboratories (Tokyo, Japan). Phosphoric acid (H_3_PO_4_), sodium hydroxide (NaOH), hydrochloric acid (HCl), sodium chloride (NaCl), potassium chloride (KCl), magnesium chloride hexahydrate (MgCl_2_·6H_2_O), calcium chloride anhydrous (CaCl_2_), and disodium sulfate (Na_2_SO_4_) were purchased from Fujifilm Wako Pure Chemical Corporation (Osaka, Japan). Dichloromethane (DCM), THF, and ethanol were purchased from Kanto Chemical Co. (Tokyo, Japan). Dodecyl bromide, piperidine, lepidine, and 3,5-di-*tert*-butyl-4-hydroxybenzaldehyde were purchased from Tokyo Chemical Industry (Tokyo, Japan).

### Microscopy

The COP mold, PCS, and CM/PCS hybrid structure were observed via field-emission SEM (SU8010, Hitachi, Ibaraki, Japan) at acceleration voltage of 10 keV. In the observation, Pt was sputtered onto each structure surface with a thickness of about 3 nm for clearer observation of the surfaces by SEM. The CM thickness was defined as the length from the bottom to top of the CM membrane in the hole and measured from the cross-sectional SEM images of the hybrid structure. The thickness was determined as the mean value in units of tens of nanometers obtained by triplicate measurement at three cross-sectional points.

### Optical characterization of the PCS

A home-built optical setup was used to measure the reflection spectra of the PCS and CM/PCS hybrid. Based on an upright microscope (Wraymer Inc., Osaka, Japan), the setup was composed of a tungsten–halogen lamp, a lens, an aperture, a half-mirror, a long-working-distance lens (Sigma Koki Co. Ltd., Hidaka, Japan), a spectrometer, and operation software (Thorlabs Inc., Newton, USA). The reflection spectrum of the PCS was measured under air conditions, and the quality factors *Q* of two spectral peaks were calculated as *Q* = FWHM/*λ*_peak_, where FWHM is the full width at a half-maximum, which was obtained by spectral peak fitting according to the Lorentz function, and *λ*_peak_ is the peak wavelength. The simulated structure was constructed based on the fabricated PCS structure, and the reflection spectrum was calculated using the FDTD method. The electric field enhancement factors *|E*^2^/*E*_0_^2^*|* were calculated at the wavelengths of peaks A and C, and their distributions were visualized.

### Optical characterization of the CM/PCS

Using the home-built optical setup, the reflection spectra of the CM/PCS hybrid structure were measured when immersed in acidic solution (0.1 M H_3_PO_4_ aq.) and basic solution (0.1 M NaOH aq.). The simulated structure was also constructed based on the fabricated CM/PCS hybrid (T_3_). The extinction coefficient of the membrane *κ*, where

$$ \kappa = \lambda A/4\pi Tln10, $$

was obtained from our previous work and applied to the simulation model, where *A* is the absorbance at a wavelength *λ* when the membrane is immersed in acidic, basic, or Ca-ion-containing sample solution (Fig. S3)^18^.

### Optical measurement of Ca ions

The responsivity of the CM/PCS hybrid to Ca ions or other cations was tested experimentally by measuring the reflection spectra of the CM/PCS hybrid structure immersed in the sample solutions. First, the CM/PCS hybrid structure was immersed in the acidic solution (0.1 M H_3_PO_4_ aq.) to protonate the dye molecules in the CM; then, it was immersed in the buffer solution (50 mM Tris–HCl, pH: 7.0) to remove the acidic solution. Next, the CM/PCS hybrid structure was immersed in the buffer solution containing Ca ions (10^−6^–10^−1^ M). The CM/PCS hybrid structure was washed using the buffer solution when the sample solutions with different concentrations of Ca ions were exchanged. Finally, the CM/PCS hybrid was immersed in the basic solution (0.1 M NaOH aq.) to deprotonate the dye molecules fully, and a structural signal was obtained to normalize the Ca ion signals. Triplicate measurement of the CM thickness for all CM/PCS hybrid structures was performed. For the other cations, Mg^2+^, Na^+^, and K^+^, the same measurements were performed in triplicate using the CM/PCS hybrid structure (T_3_).

## Supplementary information

Supplementary Information.
