# Supplementary material for: Highly sensitive optical ion sensor with ionic liquid-based colorimetric membrane/photonic crystal hybrid structure
Source: Sci Rep. 2020 Oct 7;10:16739. doi: 10.1038/s41598-020-73858-8 (PMC7542176; doi:10.1038/s41598-020-73858-8)
Supplement: Supplementary file 1 — Supplementary Information. [file 41598_2020_73858_MOESM1_ESM.docx]

**Supplementary material**

**Highly sensitive optical ion sensor with ionic liquid-based colorimetric membrane/photonic crystal hybrid structure**

Daiki Kawasaki^1^, Ryoutarou Oishi^1^, Nao Kobayashi^1^, Tatsumi Mizuta^1^,

Kenji Sueyoshi^1, 2^, Hideaki Hisamoto^1^, Tatsuro Endo^†1,^ ^2^

^1^ Osaka Prefecture University, ^2^ JST PRESTO

Corresponding author e-mail: endo@chem.osakafu-u.ac.jp

**Contents**

Text S1: Theoretical modeling of the CM/PCS hybrid structure.

Figure S1: SEM images of the PCS and CM/PCS hybrid structure.

Figure S2: Fabrication process.

Figure S3: CM extinction coefficient.

Figure S4: Optical properties of the CM/PCS hybrid structure in the acidic solution.

Figure S5: Theoretical response of the CM/PCS hybrid structure to Ca ions.

Figure S6: Response curves for different cations.

References

**Theoretical modeling of the CM/PCS hybrid**

The absorption efficiency of the dye molecules in a cavity is related to the LDOS of dipoles, which is increased by enhancing the electric field^[1, 2]^. In this study, the response of a CM/PCS hybrid to Ca ions and the absorption efficiency enhancement were theoretically calculated based on the LDOS enhancement by the PCS. The LDOS enhancement by mode A (see main text) of the dipoles at a certain position in the hole of the PCS $P_{l}$, as given by

$$\begin{aligned} P_{l}=\frac{{LDOS}_{cavity}}{{LDOS}_{free-space}}\#(S1 \end{aligned})$$

was calculated using the FDTD method, where *l* [nm] is the height above the bottom of the hole to the dipole. The LDOS enhancement factor for the dipoles in the holes was much higher than that for the dipoles outside the holes, and the LDOS enhancement of the dipoles outside the holes was negligible. Thus, the LDOS enhancement of the dipoles in the holes was evaluated. In the calculations, the dipole moment was parallel to the surface of the PCS; however, the LDOS enhancement factor for a dipole depends on its planar position and the angle of the dipole moment. Thus, to evaluate the LDOS enhancement factor for dipoles with various momentum angles in a certain plane at *l* above the bottom of a hole, a normalized function of the LDOS enhancement is defined as$P\left( l \right)$:
 $\begin{aligned} P\left( l \right)=\frac{P_{l}}{P_{l, max}} \sim\frac{P_{l}-1}{P_{l, max}-1}\#\left( S2 \right) \end{aligned}$where $P_{l, max}= P_{0}$. Due to the exponential decay of the enhanced electric field along height *l,* the LDOS enhancement factor also exponentially decreased. Thus, $P\left( l \right)$ can be described as
 $\begin{aligned} P\left( l \right)=\exp\left( -\alpha l \right)\#(S3) \end{aligned}$

where $\alpha$ is a positive constant, which describes attenuation of localized light in the holes along the height direction. Then, the enhanced absorption factor $A\left( \kappa,l \right)$ of the CM/PCS hybrid, which was defined as the integration of average absorption enhancement of dipoles in the holes, can be expressed in terms of the thickness *T*, extinction coefficient at the wavelength of peak A shown in Fig. 3 in the main text, and *κ* of the CM as
 $\begin{aligned} A\left( \kappa,T \right)= \gamma\kappa\int_{0}^{T} P\left( l \right)dl\#(S4) \end{aligned}$where $\gamma$ is an experimentally determined constant, which corrects the localized light-absorption cross-section, in other words, the interaction of dipoles in the micro-cavity due to the surrounding conditional difference of dyes between this work and previous work. Then, the normalized enhanced absorption factor $A^{'}\left( \kappa,T \right)$ can be defined as
 $\begin{aligned} A^{'}\left( \kappa,T \right)=\frac{A\left( \kappa,T \right)}{{A\left( \kappa,T \right)}_{\max}}= \gamma^{'}\kappa\left\{ 1-\exp\left( -\alpha T \right) \right\}\#\left( S5 \right) \end{aligned}$where $\gamma^{'}=\alpha/\gamma$. The reflection intensity of the CM/PCS in an acidic solution is described by *R_PCS_*, which was normalized to 1, and the extinction of light incident into the PCS is defined as $D\left( \kappa,T \right)$:
 $\begin{aligned} D\left( \kappa,T \right)=\exp\left( -\beta\kappa T \right)\#\left( S6 \right) \end{aligned}$where *β* is an experimentally determined constant, which corrects incidental light-absorption cross-section of dyes in the CM on the PCS. Then, the theoretical reflection intensity of the CM/PCS hybrid can be expressed in terms of *T* and $\kappa$ as $R\left( \kappa,T \right)$:
 $\begin{aligned} R\left( \kappa,T \right)=D\left( \kappa,T \right){\cdot R}_{PCS}\cdot\left\{ 1-A^{'}\left( \kappa,T \right) \right\}\#\left( S7 \right) \end{aligned}$

This expression can be rewritten as

$\begin{aligned} R\left( \kappa,T \right)=\exp\left( -\beta\kappa T \right)\left[ 1-\gamma^{'}\kappa\left\{ 1-\exp\left( -\alpha T \right) \right\} \right]\#\left( S8 \right) \end{aligned}$

On the other hand, the theoretical reflection intensity of the CM/Plane hybrid is described by $R_{plane}\left( \kappa,T \right)$:
 $\begin{aligned} R_{plane}\left( \kappa,T \right)=\exp\left( -\beta^{'}\kappa T \right)\#\left( S9 \right) \end{aligned}$

where $\beta'$ is an experimentally determined correction constant. Table S1 summarizes the values of the constants. These constant values were determined by applying least-squares fitting between the theoretical and experimental results.

Table S1. Coefficient values in the model.

| Constant | value |
| --- | --- |
| *α* | 0.0070 |
| *γ* | 0.024 |
| *γ*’ | 3.4 |
| *β* | 0.078 |
| *β*' | 0.0073 |


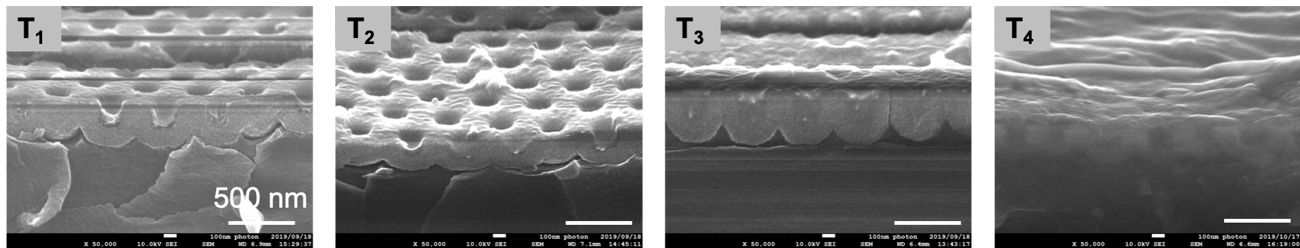


Figure S1. SEM images of the cross-sections of the CM/PCS hybrid structures. The CM thickness *T* was determined as the length from the bottom of the holes to the top of the CM components in the holes. T_1_, T_2_, T_3_, and T_4_ are 50, 100, 230, and 300 nm, respectively.

Figure S2. Fabrication of the PCS and CM/PCS hybrid structure. First, the COP mold was filled with the LPD solution for 90 min, followed by attachment of the TiO_2_ slab to the glass substrate using photo-curable resin (NOA 81). Then, it was immersed in D-limonene to demold the COP mold by dissolution of the COP polymer, and the PCS was obtained. Finally, the CM was hybridized to the PCS by spin-coating of the IL components to obtain the CM/PCS hybrid structure. In detail, see method part in main text.


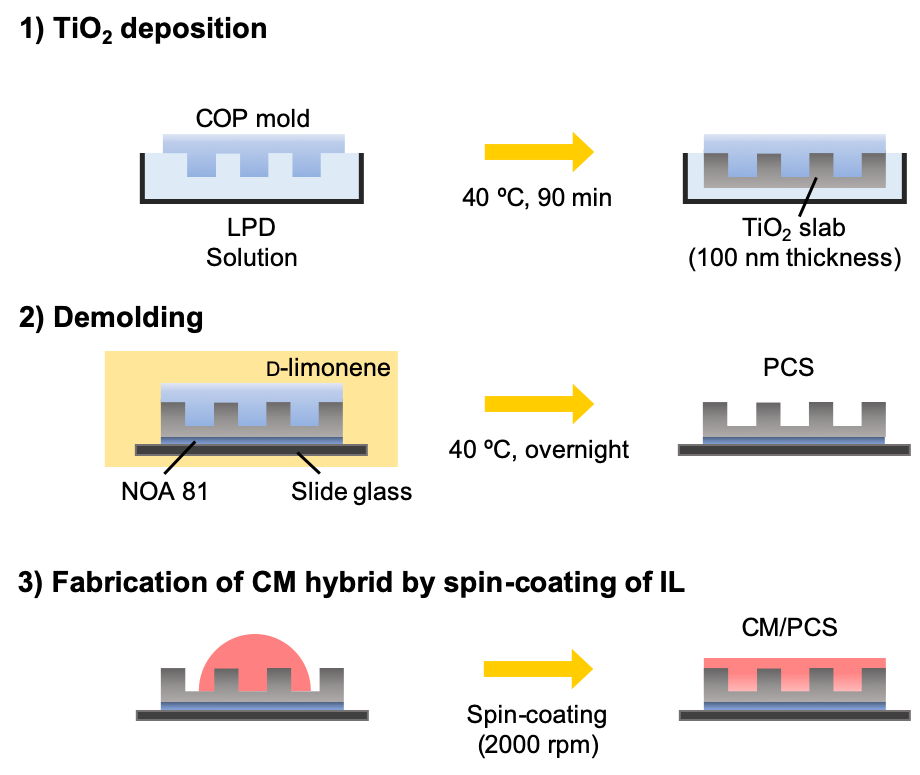

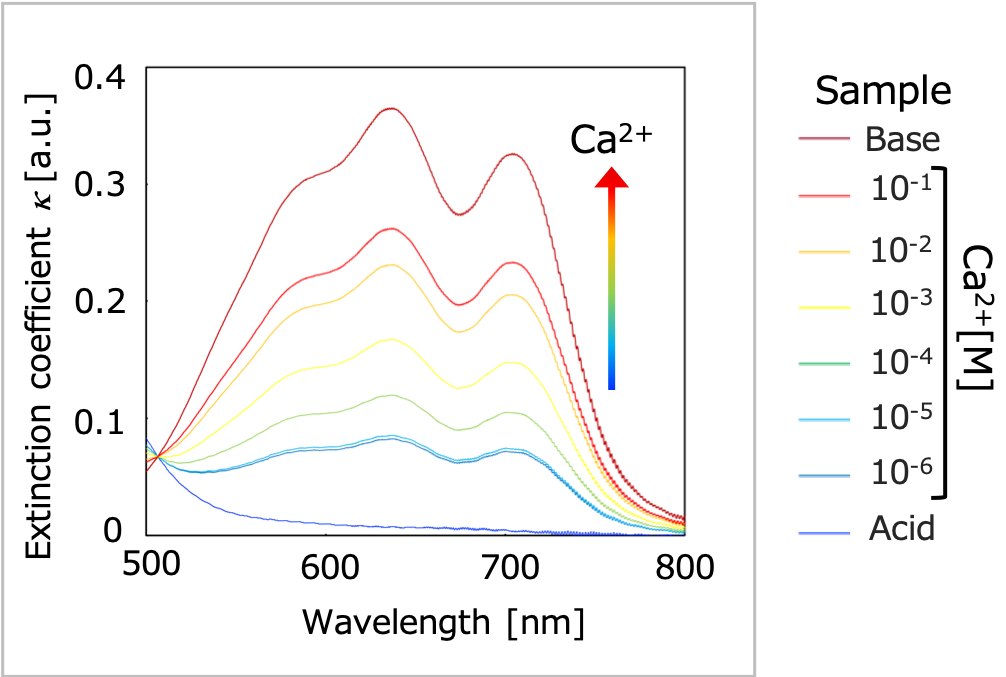


Figure S3. *κ* of the CM in different sample solutions. *κ* was obtained using $\boldsymbol{\kappa}\boldsymbol{=\lambda A/4}\boldsymbol{\pi Tln}\boldsymbol{10}$ based on our previous experimental results. *κ* increases with increasing Ca ion concentration in the wavelength range of 520–800 nm.


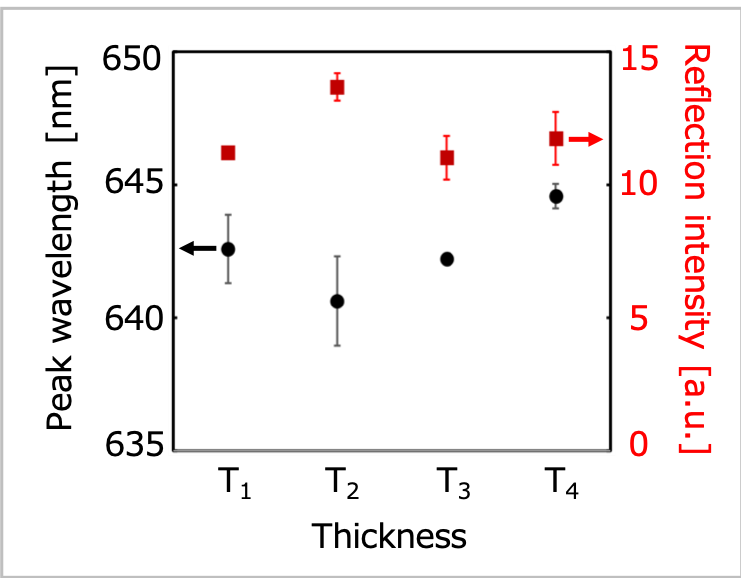


Figure S4. Optical properties of the CM/PCS hybrid structure in acidic solution for each CM thickness. The reflection intensities and peak wavelengths are independent of the CM thickness with soaking of the hybrid structures in acidic solution (i.e., without absorption by the CM). Triplicate measurement was performed (N = 3).


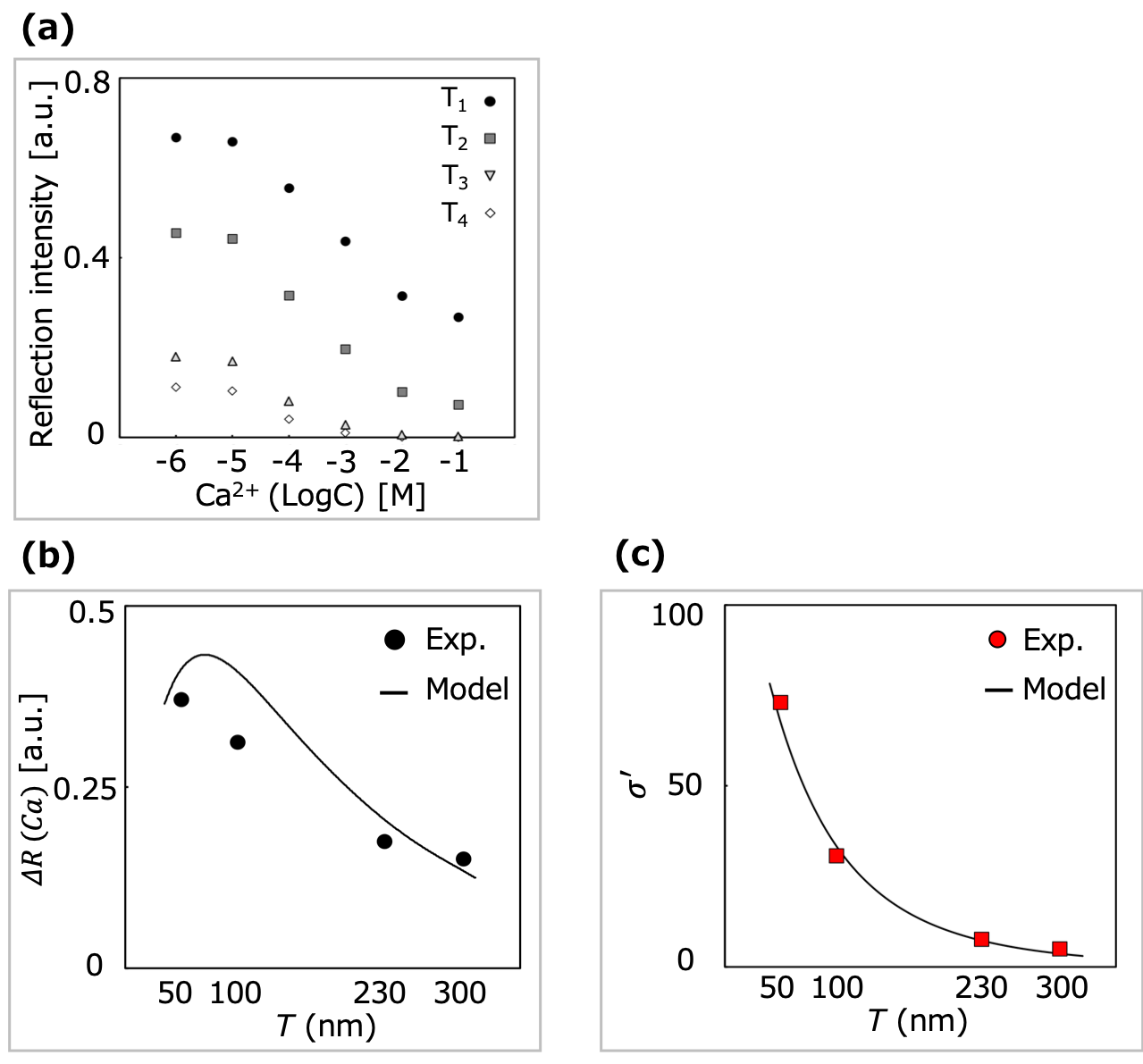


Figure S5. Theoretical modeling of the CM/PCS hybrid structure. (a) Theoretical response curves for each CM thickness. (b) Theoretical responsivity to Ca ions (solid line) and experimental results (dots). The model results show that the thickness producing a hybrid structure with the maximum responsivity is ca. 70 nm. (c) Theoretical absorption efficiency enhancement factor (solid line) and experimental results (red squares). The model and experimental results agree well.


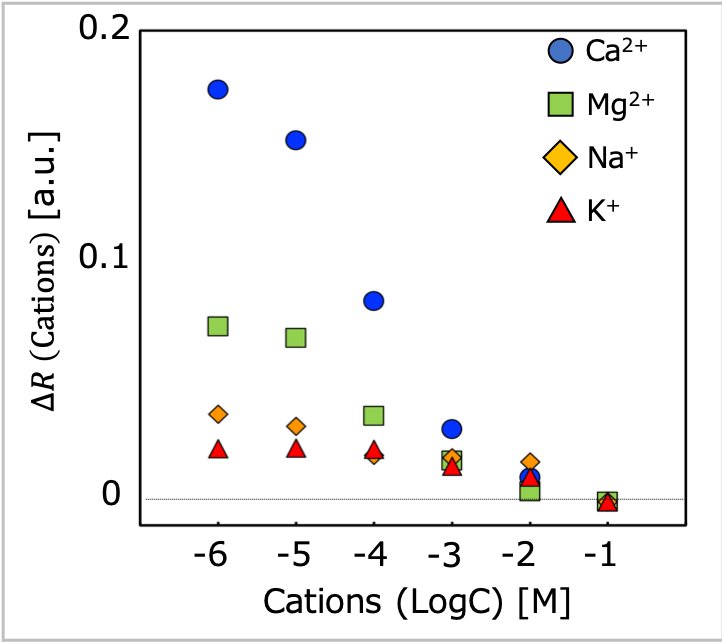


Figure S6. Response curves for different cations (Ca, Mg, Na, and K ions), which were obtained by using the CM/PCS hybrid structure with the T_3_-thickness CM. To evaluate the selectivity for different cations, the reflection intensity change $\boldsymbol{\Delta R}\left( \mathbf{Cations} \right)\boldsymbol{=R}\left( \mathbf{LogC} \right)\boldsymbol{-}\boldsymbol{R}\left( \mathbf{-}\mathbf{1} \right)$ was calculated. The experimental values are the mean values of triplicate measurements (N = 3).

**References**

1. Manga Rao, V. S. C. & Hughes, S. Single quantum-dot Purcell factor and β factor in a photonic crystal waveguide. *Phys. Rev. B* **75**, 1–7 (2007).

2. Datsyuk, V. V. Ultimate enhancement of the local density of electromagnetic states outside an absorbing sphere. *Phys. Rev. A* **75**, 1–6 (2007).
